# DuoHexaBody-CD37^®^, a novel biparatopic CD37 antibody with enhanced Fc-mediated hexamerization as a potential therapy for B-cell malignancies

**DOI:** 10.1038/s41408-020-0292-7

**Published:** 2020-04-28

**Authors:** Simone C. Oostindie, Hilma J. van der Horst, Laurens P. Kil, Kristin Strumane, Marije B. Overdijk, Edward N. van den Brink, Jeroen H. N. van den Brakel, Hendrik J. Rademaker, Berris van Kessel, Juliette van den Noort, Martine E. D. Chamuleau, Tuna Mutis, Margaret A. Lindorfer, Ronald P. Taylor, Janine Schuurman, Paul W. H. I. Parren, Frank J. Beurskens, Esther C. W. Breij

**Affiliations:** 1 Genmab, Utrecht, The Netherlands; 2 Department of Immunohematology and Blood Transfusion, Leiden University Medical Center, Leiden, The Netherlands; 3 Department of Hematology, Amsterdam University Medical Center, Amsterdam, The Netherlands; 4 Department of Biochemistry and Molecular Genetics, University of Virginia School of Medicine, Charlottesville, VA USA; 5 Present Address: Department of Immunohematology and Blood Transfusion, Leiden University Medical Center, Leiden, The Netherlands; 6 Present Address: Lava Therapeutics, Utrecht, The Netherlands

**Keywords:** Lymphoma, Drug development, Targeted therapies, Leukaemia

## Abstract

Tetraspanin CD37 has recently received renewed interest as a therapeutic target for B-cell malignancies. Although complement-dependent cytotoxicity (CDC) is a powerful Fc-mediated effector function for killing hematological cancer cells, CD37-specific antibodies are generally poor inducers of CDC. To enhance CDC, the E430G mutation was introduced into humanized CD37 monoclonal IgG1 antibodies to drive more efficient IgG hexamer formation through intermolecular Fc-Fc interactions after cell surface antigen binding. DuoHexaBody-CD37, a bispecific CD37 antibody with the E430G hexamerization-enhancing mutation targeting two non-overlapping epitopes on CD37 (biparatopic), demonstrated potent and superior CDC activity compared to other CD37 antibody variants evaluated, in particular ex vivo in patient-derived chronic lymphocytic leukemia cells. The superior CDC potency was attributed to enhanced IgG hexamerization mediated by the E430G mutation in combination with dual epitope targeting. The mechanism of action of DuoHexaBody-CD37 was shown to be multifaceted, as it was additionally capable of inducing efficient antibody-dependent cellular cytotoxicity and antibody-dependent cellular phagocytosis in vitro. Finally, potent anti-tumor activity in vivo was observed in cell line- and patient-derived xenograft models from different B-cell malignancy subtypes. These encouraging preclinical results suggest that DuoHexaBody-CD37 (GEN3009) may serve as a potential therapeutic antibody for the treatment of human B-cell malignancies.

## Introduction

B-cell malignancies comprise a heterogeneous group of lymphoproliferative disorders including non-Hodgkin lymphomas (NHL) and chronic lymphocytic leukemia (CLL). In addition to chemotherapy and small molecule inhibitors, immunotherapy with anti-CD20 monoclonal antibodies (mAbs), such as rituximab, ofatumumab, and obinutuzumab, has significantly improved the outlook for patients with B-NHL and CLL^1–3^. However, many patients eventually relapse and become resistant to treatment, creating an unmet need for alternative therapeutic strategies. In recent years, the tetraspanin plasma membrane protein CD37 has gained renewed interest as a promising therapeutic target for B-cell malignancies^4–7^. CD37 is selectively expressed on mature B cells and has limited or no expression on other hematopoietic cells such as T cells and NK cells, granulocytes, monocytes and dendritic cells^8–10^.

CD37 is involved in the spatial organization of the B-cell plasma membrane by forming tetraspanin-enriched micro domains (TEMs) through lateral associations with interaction partners, such as other tetraspanins or integrins^11,12^. CD37 is signaling-competent as it contains intracellular functional ITIM-like and ITAM-like motifs that play a role in pro-survival and pro-apoptotic signaling via the PI3K/AKT pathway. In addition, it controls IL-6 receptor signaling through interaction with SOCS3^12,13^.

In cancer, CD37 is highly expressed on malignant B cells in a variety of B-cell lymphomas and leukemias, including NHL and CLL^14,15^. To date, multiple CD37-targeting agents have shown preclinical or clinical efficacy^5–7^, including antibody drug conjugates^16,17^, a small modular immuno-pharmaceutical protein (SMIP)^18^, an antibody with enhanced antibody-dependent cellular cytotoxicity (ADCC) capacity^19^, a radiolabeled antibody^20^ and chimeric antigen receptor (CAR) T cells^21^. The effector mechanisms of these agents include direct cytotoxicity mediated through conjugated cytotoxic or radioactive payloads, classical FcγR-mediated effector functions such as ADCC, and T-cell mediated cytotoxicity. Interestingly, CD37 antibody-based therapeutics currently in (pre-)clinical development are poor inducers of complement-dependent cytotoxicity (CDC)^5–7^, another powerful Fc-mediated effector mechanism for killing hematological cancer cells^22,23^.

We have previously reported that activation of the classical complement pathway by IgG antibodies depends on IgG hexamer formation upon binding to membrane bound antigens. IgG hexamers, which form through intermolecular Fc-Fc interactions, provide an optimal docking site for hexavalent C1q^24–26^. Activation of C1 triggers the complement cascade involving a series of proteolytic events leading to formation of membrane attack complexes that eventually kill target cells via disruption of their cell membrane. Introduction of a single point mutation, such as E430G, in the IgG Fc domain increases IgG hexamer formation and enhances CDC activity^27,28^. We combined this approach with the bispecific antibody technology DuoBody^®^ to generate an obligate bispecific antibody for which potency was further increased compared to combinations of the parent molecules. Obligate bispecific antibodies represent a novel and most promising concept in current therapeutic antibody drug development^29,30^.

We hereby report the generation of a panel of CD37-targeting mAbs with an E430G hexamerization-enhancing mutation and characterized the preclinical mechanism of action and anti-tumor activity of the single mAbs, mAb combinations and CD37 biparatopic (bispecific) antibodies. It was demonstrated that CDC efficacy by single CD37-targeting mAbs was enhanced by combining two non-cross-blocking mAbs, which was most evident in the context of a biparatopic antibody variant, DuoHexaBody-CD37. DuoHexaBody-CD37 also induced potent FcγR-mediated effector functions, including ADCC and antibody-dependent cellular phagocytosis (ADCP). In addition, DuoHexaBody-CD37 showed significant anti-tumor efficacy in vivo in human cell line- and patient-derived xenograft models, indicating that DuoHexaBody-CD37 may serve as a promising novel therapeutic antibody for treatment of human B-cell malignancies.

## Materials and methods

### Antibodies

Anti-CD37 antibodies were generated through immunization (MAB Discovery GmbH, Germany) of rabbits with a mixture of HEK293F cells expressing human (sequence no. NP_001765) or cynomolgus monkey (Macaca fascicularis, sequence no. XP_005589942) CD37 or a mixture of Fc-fusion proteins containing the large extracellular loop of human or cynomolgus monkey CD37. CD37 antibodies were produced recombinantly as chimeric human IgG1s containing the hexamerization-enhancing mutation E430G (HexaBody^®^ molecules^27^) and F405L or K409R mutations for bispecific antibody generation by controlled Fab-arm exchange (cFAE; DuoBody technology^30,31^) as appropriate. Humanized antibody sequences were generated using CDR-grafting in optimized human germ-line variable region sequences at Abzena (Cambridge, UK). The anti-HIV-1 gp120 mAb IgG1-b12 was used as an negative control antibody (IgG1-ctrl)^32^. Rituximab (MabThera^®^), ofatumumab (Arzerra^®^) and obinutuzumab (Gazyva^®^) were commercially obtained.

### Cell lines, patients, donors, and reagents

Details on cell lines used in this study are summarized in online Supplementary Table S1. All primary patient cells were obtained after written and informed consent and stored using protocols approved by institutional review boards in accordance with the declaration of Helsinki (see Online Supplementary Information). Blood samples and buffy coats from healthy human donors were obtained from the University Medical Center Utrecht (Utrecht, The Netherlands) and Sanquin (Amsterdam, The Netherlands), respectively. Pooled normal human serum (NHS; AB positive) was obtained from Sanquin. Details on antibodies/reagents used to define cell subsets within samples used for flow cytometry are provided in Online Supplementary Tables S2–S5.

### Antibody binding assays

Antibody binding was assessed using target cells incubated with antibody for 30 min at 4 °C. After washing, cells were incubated with R-Phycoerythrin (PE)-conjugated goat-anti-human IgG F(ab’)2 (Jackson ImmunoResearch Laboratories, West Grove, PA, USA) for 30 min at 4 °C. Cells were washed and binding was analyzed by determining the geometric mean fluorescensce intensity (gMFI) of the PE signal using flow cytometry.

For binding competition assays, target cells were incubated with primary unlabeled antibodies (final concentration 20 µg/ml) for 15 min at room temperature. Next, Alexa Fluor 488 (A488)-labelled antibodies (by reaction with N-hydroxysuccinimidyl-esters following manufacturer’s instructions [Molecular Probes, Eugene, OR, USA]) were added to cells at final concentration of 2 µg/ml, followed by incubation for 15 min at room temperature. Cells were washed and gMFI of the A488 signal was determined by flow cytometry.

### Alanine scanning

A CD37 single residue alanine library was generated (GeneArt, Regensburg, Germany) in which all amino acid residues in the extracellular domains of human CD37 (UniProt P11049) were individually mutated to alanine, except for cysteines. The library was used to map amino acids in the extracellular loops of human CD37 involved in binding of mAbs Hx-CD37-010 and Hx-CD37-016 (details summarized in Online Supplementary Information).

### CDC assays

CDC assays were performed as described using tumor cells incubated with antibody for 45 minutes at 37 °C in the presence of NHS (20% final concentration) as a complement source^33^.

### Expression analysis

Expression levels of cellular markers were determined as described in ref. ^33^ using an indirect immunofluorescence assay (QIFIKIT^®^, Agilent, Santa Clara, CA, USA) according to the manufacturer’s instructions.

### ADCC and ADCP assays

Activation of FcγRIIa- (H-131) and FcγRIIIa-mediated (V-158) intracellular signaling was quantified using Luminescent Reporter Bioassays (Promega, Madison, WI, USA), according to the manufacturer’s recommendations. Chromium-51 (Cr^51^) release ADCC assays were performed as described^34^ and summarized in Online Supplementary Information. ADCP assays were performed using tumor cells labeled with calcein AM (Life Technologies, Carlsbad, CA, USA) or pHRodo^TM^ Red AM Intracellular pH Indicator (ThermoFisher Scientific, Waltham, MA, USA) according to the manufacturer’s instructions and opsonized with antibodies for 15 min at 37 °C. Human monocyte-derived macrophages (h-MDM, isolation and culturing detailed in Online Supplementary Information) were added at effector to target (E:T) ratios of 2:1 or 1:1 and incubated for 4 h at 37 °C/5%CO_2_. During incubation, images were captured using an IncuCyte S3 Live Cell Analysis System with a ×10 objective lense and acquired/processed using IncuCyte S3 software. Alternatively, tumor cells and h-MDM were stained for surface markers after incubation using fluorochrome-conjugated antibodies for 30 min at 4 °C, fixed using 4% paraformaldehyde (ChemCruz, Dallas, TX, USA) and analyzed by flow cytometry. CD11b^+^/calcein AM^+^/CD19^−^ cells were defined as h-MDM that phagocytosed Daudi cells. CD11b^−^/calcein AM^+^ cells were defined as non-phagocytosed Daudi cells remaining after co-culture, used to determine target cell depletion.

### Whole-blood assays

Binding and cytotoxicity assays were performed with heparin- and hirudin-treated blood samples from healthy human donors, respectively. For cytotoxicity, blood samples were incubated with antibody for 4 h at 37 °C. Next, red blood cells were lysed and samples were stained for 30 min at 4 °C with fluorochrome-labeled lineage-specific antibodies and TO-PRO-3 to characterize cell subsets and dead or dying cells respectively. For binding, red blood cells were first lysed and subsequently incubated with designated antibody mixtures. Binding was assessed by flow cytometry and expressed as the gMFI of AF488 fluorescence intensity for viable cell subsets. Depletion was determined as:

$${\mathrm{\% }}\,{\mathrm{target}}\,{\mathrm{cell}}\,{\mathrm{depletion}} = 100\, \times \,\left( {\frac{{{\mathrm{fraction}}\,{\mathrm{live}}\,{\mathrm{B}}\,{\mathrm{cells}}\,{\mathrm{no}}\,{\mathrm{Ab}}\,{\mathrm{ctrl}} - {\mathrm{fraction}}\,{\mathrm{live}}\,{\mathrm{B}}\,{\mathrm{cells}}\,{\mathrm{sample}}}}{{{\mathrm{fraction}}\,{\mathrm{live}}\,{\mathrm{B}}\,{\mathrm{cells}}\,{\mathrm{no}}\,{\mathrm{Ab}}\,{\mathrm{ctrl}}}}} \right)$$

### Animal studies

Cell line-derived xenograft (CDX) and patient-derived xenograft (PDX) studies were conducted following protocols approved by institutional ethical committees, as provided in Online Supplementary Information and Supplementary Table S6. In vivo pharmacokinetic analysis was performed as described in ref. ^27^.

### Data processing

Flow cytometry data were analyzed using FlowJo V10 software. Graphs were plotted and analyzed using GraphPad Prism 8.0. Dose-response curves were generated using best-fit values of non-linear dose-response fits using log-transformed concentrations. All data shown are representative of at least two independent replicate experiments. Statistical differences in median animal tumor volumes were compared between treatment groups on the last day all groups were complete. In case of equal variance between groups (Bartlett’s test) the parametric One Way ANOVA Uncorrected Fisher’s LSD test (Daudi-Luc) was used. In case of unequal variance between groups (Bartlett’s test) the non-parametric Mann-Whitney test (JVM-3, DOHH-2, NHL PDX) was used.

## Results

### Generation of CD37 mAbs and analysis of their binding characteristics

**Fig. 1:**
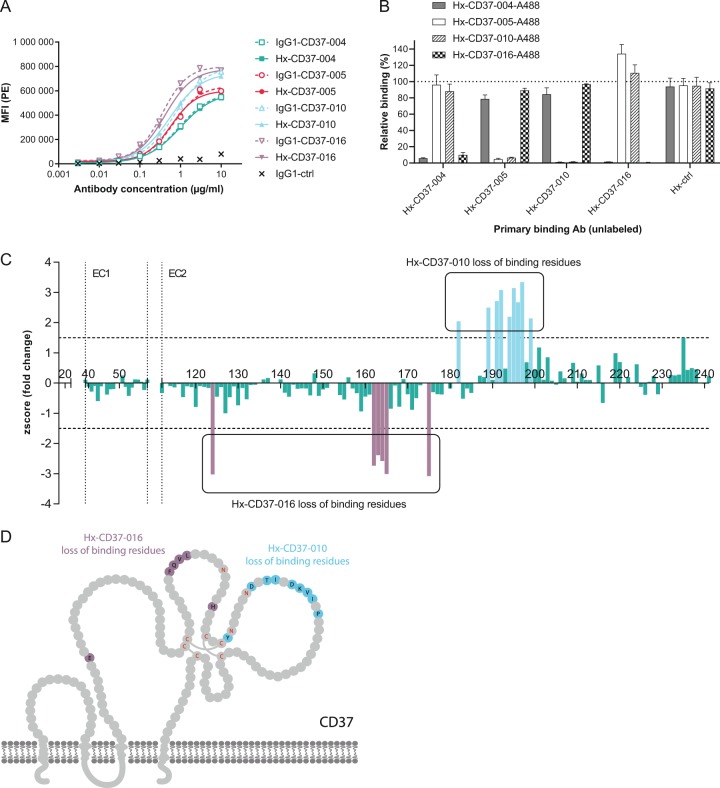
Binding characteristics of CD37 panel mAbs. **a** Binding of WT (IgG1) and hexamerization-enhanced (Hx) antibody variants of the CD37 panel to Daudi cells. Antibody binding was assessed by flow cytometry and is expressed as the gMFI of the PE signal from a secondary IgG detection antibody. **b** Binding competition between Hx-CD37 panel antibodies was assessed on Raji cells by pre-incubating cells with 20 µg/ml unlabeled Hx-CD37 panel antibodies (primary binding) followed by incubation with A488-labeled antibodies (2 µg/ml). Relative binding in presence of competing antibody was plotted (percentage relative binding = [gMFI A488 in presence of competing antibody]/[gMFI A488 in absence of competing antibody] × 100). Data represents the mean and standard deviation (SD) of triplicate measurements. **c** Mapping of amino acids in the extracellular loops of human CD37 involved in binding of Hx-CD37-010 or Hx-CD37-016 as determined by alanine scanning. A z-score (fold change in binding compared to binding of a control antibody) for each mutant position was calculated and plotted. *Z*-scores < 0 indicate loss of binding of Hx-CD37-016 in comparison to Hx-CD37-010 while *z*-scores > 0 indicate loss of binding of Hx-CD37-010 in comparison to Hx-CD37-016. Amino acid residues where the z-score was higher than 1.5 (Hx-CD37-010) or lower than –1.5 (Hx-CD37-016), indicated by the horizontal dotted lines, were considered as “loss of binding mutants”. The number above the x-axis refers to amino acid positions in full length human CD37. EC1 = small extracellular loop, EC2 = large extracellular loop of CD37, respectively. **d** Amino acid residues involved in Hx-CD37-010 (blue) and Hx-CD37-016 (purple) binding to CD37 are depicted in a graphical representation based on UniProtKB P11049. The extracellular domain of CD37 contains six cysteines (Cys, C), which are indicated in red; these form intramolecular disulphide bonds and contribute to the conformation of the tetraspanin. The asparagine residues (Asn, N) in the *N*-glycosylation site consensus sequence Asn-Xxx-Ser (NXS) are indicated in red.

Rabbits were immunized with CD37 antigen to generate a diverse panel of mAbs recognizing human CD37. After humanization using CDR-grafting, antibodies were expressed in a human IgG1 backbone with and without the E430G hexamerization-enhancing mutation (Hx) and binding to CD37 on human tumor cells was assessed using Daudi cells. Four humanized CD37 mAbs were selected for further studies based on specific and efficient target binding (EC_50_ < 0.1 µg/ml), CDR sequence diversity and cross-reactivity with human and cynomolgus monkey CD37. Hx-CD37-004, Hx-CD37-005, Hx-CD37-010 and Hx-CD37-016, and corresponding wild-type (WT) IgG1 variants showed dose-dependent binding to Daudi cells (Fig. 1a) with EC_50_ values ranging from 0.42 to 0.92 µg/ml. Comparable binding of WT anti-CD37 mAbs indicated that binding was not affected by the E430G mutation. We next performed cross-block binding experiments on Raji cells to examine cross-competition between the four humanized CD37 mAbs. Hx-CD37-004 competed with CD37-016 for binding, and Hx-CD37-005 competed with Hx-CD37-010 for binding to CD37 (Fig. 1b). The mutually competing antibodies Hx-CD37-004 and Hx-CD37-016 were able to simultaneously bind to CD37 with one of the other mutually competing antibodies Hx-CD37-005 or Hx-CD37-010, thereby indicating that the four antibodies represent two different cross-blocking groups.

We selected one mAb candidate from each cross-blocking group, Hx-CD37-010 and Hx-CD37-016, and used alanine scanning analysis to map epitopes within the extracellular loops of human CD37. A library with alanine substitutions at all extracellular residues of human CD37, except for cysteines, was generated. Alanine mutants were expressed individually in HEK293F™ cells and binding of Hx-CD37-010 and Hx-CD37-016 was determined by flow cytometry. Loss of Hx-CD37-010 binding to human CD37 was observed with alanine substitutions at position Y182, D189, T191, I192, D194, K195, V196, I197, and P199, while for Hx-CD37-016, loss of antibody binding was observed with alanine substitutions at position E124, F162, Q163, V164, L165 and H175 (Fig. 1c). These results showed that residues identified to be crucial for binding of Hx-CD37-010 are distinct from residues crucial for binding of Hx-CD37-016. Together, the binding analyses demonstrate that Hx-CD37-010 and Hx-CD37-016 bind different residues within the second extracellular domain (EC2) of human CD37 (Fig. 1d).

### CDC activity of CD37 mAbs is potentiated by enhanced hexamerization and dual epitope targeting

**Fig. 2:**
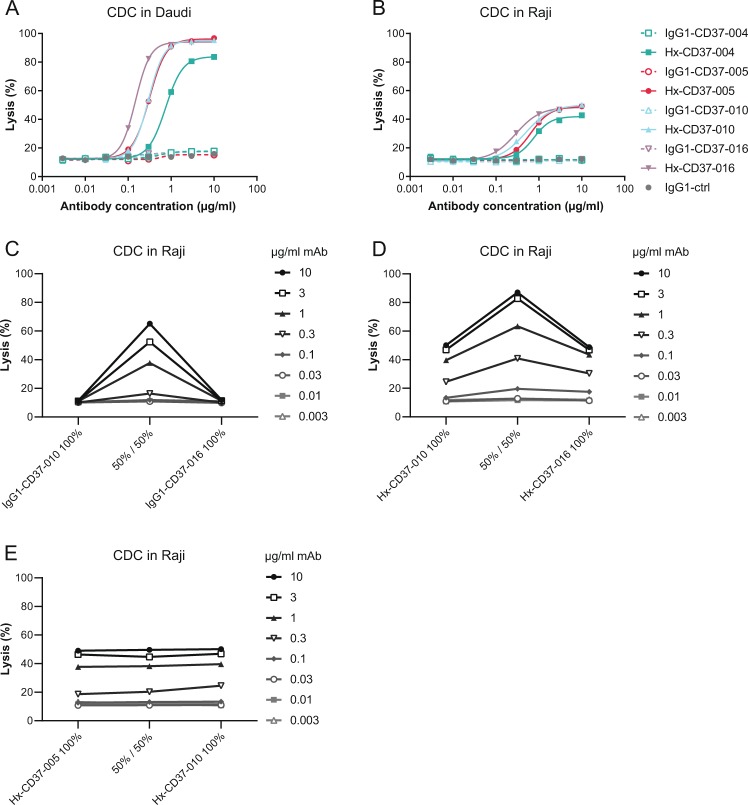
CDC activity of CD37 mAbs is potentiated by enhanced hexamerization and dual epitope targeting. **a**, **b** CDC in Daudi (**a**) and Raji (**b**) cells opsonized with WT (IgG1) or hexamerization-enhanced (Hx) variants of CD37 mAbs. **c**, **d** CDC in Raji cells opsonized with combinations of non-cross-blocking IgG1-CD37 (**c**) or Hx-CD37 (**d**) mAbs. **e** CDC in Raji cells opsonized with combinations of cross-blocking Hx-CD37 mAbs. The indicated antibody concentrations represent the total antibody concentration used. CDC induction was assessed in the presence of 20% NHS and expressed as the percentage lysis determined by the fraction of PI-positive cells.

We previously reported that CDC by CD37 mAbs is potentiated by introducing the hexamerization-enhancing mutation E430G in the IgG Fc domain^33^ and therefore investigated the potency of the novel CD37 mAbs to induce CDC in vitro. Whereas WT IgG1-CD37 antibodies were inactive, hexamerization-enhanced variants induced dose-dependent and potent CDC in Daudi cells (EC_50_ ranging from 0.15 to 0.75 µg/ml) (Fig. 2a). In addition, introduction of the E430G mutation unlocked CDC activity of the CD37 mAbs in Raji cells (EC_50_ ranging from 0.29 to 0.77 µg/ml), which are expected to be less sensitive to CDC due to higher expression of complement regulatory protein CD59 (Fig. 2b)^35^. An alternative way to enhance CDC is by dual epitope targeting using non-cross-blocking antibody combinations, as has been previously reported for a number of cell surface antigens, for example EGFR^36^. Also here, the WT IgG1-CD37 mAbs did not induce CDC as single agents, whereas combinations of non-cross-blocking WT IgG1-CD37-010 and IgG1-CD37-016 potentiated CDC to 65% maximum lysis in Raji cells (Fig. 2c). Interestingly, while hexamerization-enhanced variants of these non-cross-blocking CD37 mAbs individually induced 50% maximum lysis in Raji cells, CDC-mediated lysis was strongly enhanced in the combination (87% maximum lysis) (Fig. 2d), thereby clearly outperforming combinations of the WT non-cross-blocking CD37 mAbs. These results were confirmed with combinations of the other CD37-specific non-cross-blocking mAbs (data not shown). In contrast, combinations of Hx-CD37 mAbs that compete for CD37 binding did not show enhanced CDC activity compared to single antibodies, including the combination of Hx-CD37-005 and Hx-CD37-010 (Fig. 2e) and the mixture of Hx-CD37-004 and Hx-CD37-016 (data not shown). These results indicate that CDC activity of the CD37 mAbs is potentiated by enhanced hexamerization through the E430G mutation and by dual epitope targeting.

### A biparatopic hexamerization-enhanced CD37 antibody variant, DuoHexaBody-CD37, induces superior CDC activity in vitro and ex vivo

**Fig. 3:**
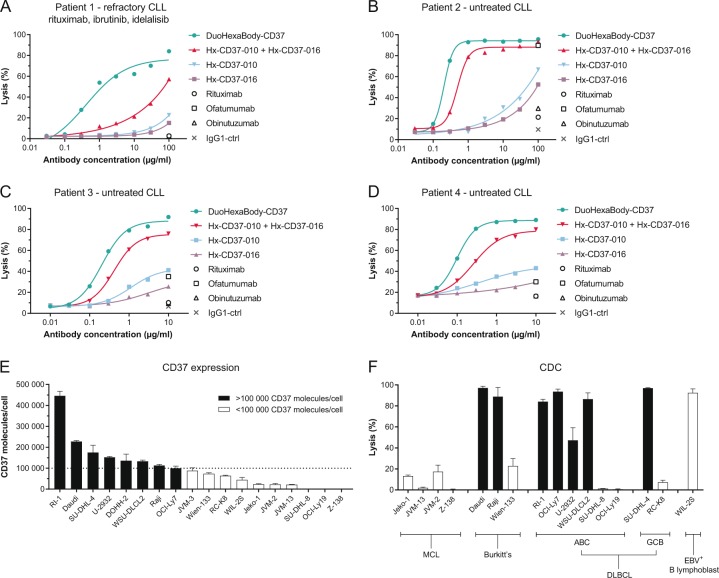
A biparatopic hexamerization-enhanced CD37 antibody variant, DuoHexaBody-CD37, induces superior CDC activity in vitro and ex vivo. **a**–**d** PBMCs (**a**, **b**) or purified B cells (**c**, **d**) from four chronic lymphocytic leukemia (CLL) patients were used to determine CDC induction by DuoHexaBody-CD37, and Hx-CD37 antibody variants (Hx-CD37-010 and Hx-CD37-016) alone or in combination (1:1 equimolar mixture). CDC induction was assessed in the presence of 20% NHS. The percentage lysis was determined by the fraction of PI-positive (**a**, **b**) or 7AAD-positive cells (**c**, **d**). **e** 17 B-lymphoma cell lines and the Epstein-Barr virus (EBV)-positive B-lymphoblastic cell line WIL-2S were evaluated for CD37 molecule expression on the cell surface using quantitative flow cytometry. The dotted line indicates 100,000 CD37 surface molecules/cell. **f** The capacity of 10 µg/ml DuoHexaBody-CD37 to induce CDC in the presence of 20% NHS was tested in 16 cell lines also described in **e** and expressed as the percentage lysis determined by the fraction of PI-positive cells. The cell lines are grouped per B-lymphoma subtype: mantle cell lymphoma (MCL), Burkitt’s lymphoma, diffuse large B-cell lymphoma (DLBCL) with activated B-cell (ABC) or germinal center B-cell (GCB) subtypes and an EBV-positive B-lymphoblastic cell line. Cell lines with >100,000 CD37 molecules per cell are indicated by black bars, and those with <100,000 CD37 molecules per cell by white bars. Either four (**e**) or two (**f**) replicates were used per cell line (mean ± SD).

We next explored the possibility of combining enhanced hexamerization with dual epitope targeting in a bispecific antibody. A biparatopic CD37 mAb variant, DuoHexaBody-CD37, was generated through controlled Fab-arm exchange between Hx-CD37-010 and Hx-CD37-016^30,31^. The CDC activity of DuoHexaBody-CD37 was compared to that of single mAbs and mAb combinations in samples from untreated CLL patients and a CLL patient relapsed/refractory to rituximab, ibrutinib and idelalisib. Strikingly, DuoHexaBody-CD37 induced superior CDC activity in all patient-derived CLL samples compared to either the single mAbs or the combination, which was most apparent in the refractory CLL sample (Fig. 3a–d). Consistent with results in tumor cell lines, the mAb combination showed enhanced CDC compared to the single mAbs. Comparison of DuoHexaBody-CD37 with the approved CD20 antibodies rituximab, ofatumumab and obinutuzumab demonstrated superior CDC in all patient samples tested. None of the approved CD20 antibodies induced CDC in the refractory CLL sample (Fig. 3a) while in untreated CLL samples, only ofatumumab induced CDC at concentrations of 10 (Fig. 3c, d) and 100 μg/ml (Fig. 3b).

The capacity of DuoHexaBody-CD37 to induce CDC in malignant B cells was further confirmed in CDC assays in vitro using 16 tumor cell lines with varying CD37 expression levels, derived from different B-cell lymphoma subtypes (Fig. 3e). DuoHexaBody-CD37 induced potent CDC in 8 of the 16 cell lines tested, with generally higher levels of tumor cell lysis observed in cell lines with CD37 expression levels above 100,000 copies/cell (Fig. 3e, f).

### DuoHexaBody-CD37 induces efficient ADCC and ADCP in vitro

**Fig. 4:**
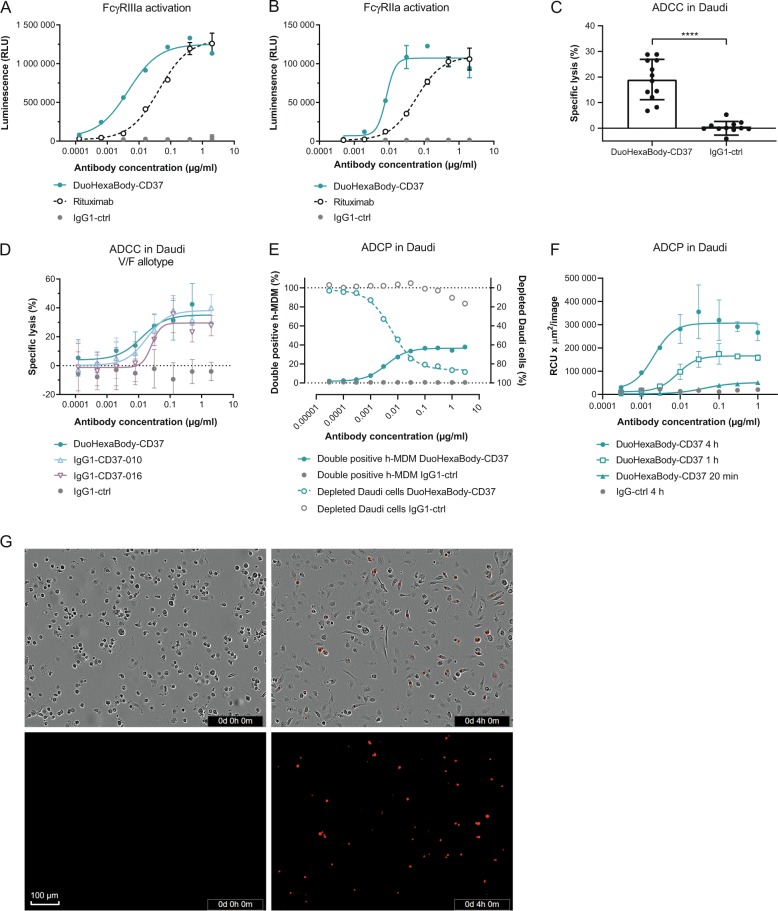
DuoHexaBody-CD37 induces efficient ADCC and ADCP in vitro. **a**, **b** FcγRIIIa (**a**) and FcγRIIa (**b**) crosslinking by DuoHexaBody-CD37 was analyzed in a bioluminescent Reporter Bioassay using Daudi target cells and engineered FcγRIIIa- or FcγRIIa-expressing Jurkat effector T cells that express luciferase upon FcγR crosslinking. Luciferase production is presented as relative luminescence units (RLU). Error bars represent the mean ± SD of duplicate measurements. **c** ADCC by 2 µg/ml DuoHexaBody-CD37 was evaluated in a classical ^51^Cr release assay using Daudi target cells and PBMCs from 12 healthy human donors as a source of effector cells (E:T of 100:1). The percentage lysis was calculated relative to a Triton X-100 control (100% lysis) and no antibody control (0% lysis). ****P < 0.00001, paired *T*-test with two-tailed 95% confidence intervals. **d** Dose-response ADCC (mean percentage lysis ± SD of three replicate samples) induced by DuoHexaBody-CD37, WT IgG1-CD37-010 and WT IgG1-CD37-016 shown for one representative responsive donor as described in **c**. Error bars represent the mean ± SD of triplicate measurements. **e** ADCP induced by DuoHexaBody-CD37 using Daudi target cells and monocyte-derived h-MDM from healthy human donors as a source of effector cells. Calcein AM-labeled Daudi cells opsonized with DuoHexaBody-CD37 were incubated with CD11b + h-MDM at an E:T ratio of 2:1 and ADCP was analyzed by flow cytometry after a 4 h co-culture. The amount of h-MDMs that phagocytosed Daudi cells is presented as percent CD11b+/calcein AM+/CD19^-^ double positive cells. CD19 was used to exclude macrophages with bound instead of phagocytosed tumor cells. The percentage CD11b^−^/calcein AM+ cells was determined as an indicator of the amount of non-phagocytosed Daudi cells; presented here as a depleted cell fraction relative to a no antibody control sample. Data from one representative donor out of three is shown. **f** ADCP of pHRodo-labeled Daudi target cells by h-MDM induced by DuoHexaBody-CD37 over time at an E:T ratio of 1:1, shown for one out of three representative donors. Red fluoresence indicates phagocytosed Daudi target cells by h-MDM. ADCP was quantified by the total sum of the red fluorescent intensity in the image (RCUxµm^2^/image) and presented as the mean ± SD of duplicate measurements. **g** Phase contrast- and red fluorescent images of DuoHexaBody-CD37-opsonized (1 µg/ml) pHRodo-labeled Daudi target cells co-cultured with h-MDM effector cells at 0 and 4 h incubation as described in **f**.

While the primary rationale behind the development of DuoHexaBody-CD37 was focused on maximizing its capacity to induce CDC, other Fc-mediated effector functions such as ADCC and ADCP, known to contribute to tumor cell kill, were also tested. The potential of DuoHexaBody-CD37 to induce ADCC and ADCP was first evaluated in FcγRIIIa (V-158) and FcγRIIa (H-131) reporter assays, respectively. When bound to Daudi target cells, DuoHexaBody-CD37 induced efficient, dose-dependent activation of FcγRIIIa and FcγRIIa signaling in transfected Jurkat effector T cells (Fig. 4a, b). FcγRIIIa and FcγRIIa signaling was at least comparable to that induced by the CD20 antibody rituximab.

ADCC induction by DuoHexaBody-CD37 was further evaluated in a ^51^Cr release assay using Daudi cells as target cells and human PBMCs from 12 healthy human donors as effector cells (E:T ratio 100:1). DuoHexaBody-CD37 induced efficient, dose-dependent ADCC of Daudi cells (EC_50_ = 9.9 ± 10.0 ng/ml), which was comparable to the WT IgG1-CD37 mAbs without the E430G and F405L/K409R mutations (Fig. 4c, d).

Next, ADCP was assessed in a flow cytometry-based assay using calcein AM-labeled Daudi target cells and h-MDM as effector cells. DuoHexaBody-CD37 induced efficient phagocytosis of calcein AM-labeled Daudi target cells by h-MDM, as illustrated by a dose-dependent increase in CD11b^+^/calcein AM^+^ double positive h-MDM (EC_50_ = 13.5 ± 13.1 ng/ml), resulting in almost complete depletion of Daudi target cells (EC_50_ = 5.8 ± 2.3 ng/ml) (Fig. 4e). DuoHexaBody-CD37-mediated ADCP was confirmed in an image-based assay using Daudi cells labeled with the pH-sensitive pHRodo dye that becomes increasingly fluorescent in the acidic lysosomal environment. Also here, DuoHexaBody-CD37 induced efficient engulfment and lysosomal degradation of Daudi cells by h-MDM (Fig. 4f, g). Together, these data demonstrate that DuoHexaBody-CD37 induces efficient FcγR-mediated immune effector functions to kill CD37-positive tumor cells.

### DuoHexaBody-CD37 depletes B cells, but not other leukocyte populations in human whole blood

**Fig. 5:**
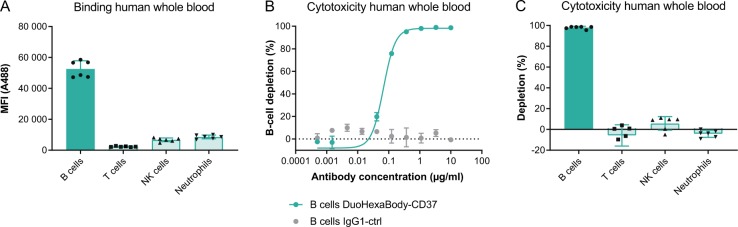
DuoHexaBody-CD37 depletes B cells, but not other leukocyte populations in human whole blood. Binding to- and depletion of different leukocyte cell subsets in human whole blood by DuoHexaBody-CD37 was assessed by flow cytometry using six healthy donors. **a** Binding of A488-labeled DuoHexaBody-CD37 (30 µg/ml) is expressed as the gMFI ± SD of the A488 signal. **b**, **c** Cell depletion induced by a concentration series (**b**, one representative donor) or 10 µg/ml (**c**, six donors) DuoHexaBody-CD37 after a four hour incubation period presented as mean percentage depletion ± SD relative to a no antibody control sample. Leukocyte subsets were characterized as: B cells (CD19^+^), T cells (CD3^+^), NK cells (CD56+) and neutrophils (CD66^+^/CD16^+^).

CD37 is reported to be highly expressed on mature B cells, with low expression levels on other leukocyte subsets^8–10^. Since non-malignant B-cell depletion may be used as a safety- and pharmacodynamic biomarker when exploring B-cell targeting therapies, the ability of DuoHexaBody-CD37 to bind and deplete B cells versus other leukocyte subsets was evaluated in whole-blood derived from six healthy human donors. The blood was hirudin anticoagulated to preserve complement activity. DuoHexaBody-CD37 showed efficient binding to CD19^+^ B cells, while low binding was observed to T cells, NK cells, and neutrophils for all six healthy human donors tested (Fig. 5a). DuoHexaBody-CD37 showed potent depletion of the CD19^+^ B-cell population compared to the negative control, with 98% ± 1.3% depletion at 10 µg/ml and an average EC_50_ of 0.077 ± 0.039 µg/ml (Fig. 5b). For the T cells, NK cells, and neutrophils that showed low DuoHexaBody-CD37 binding, no depletion was observed at saturating mAb concentrations of 10 µg/ml (Fig. 5c).

### DuoHexaBody-CD37 shows anti-tumor activity in vivo in xenograft models

**Fig. 6:**
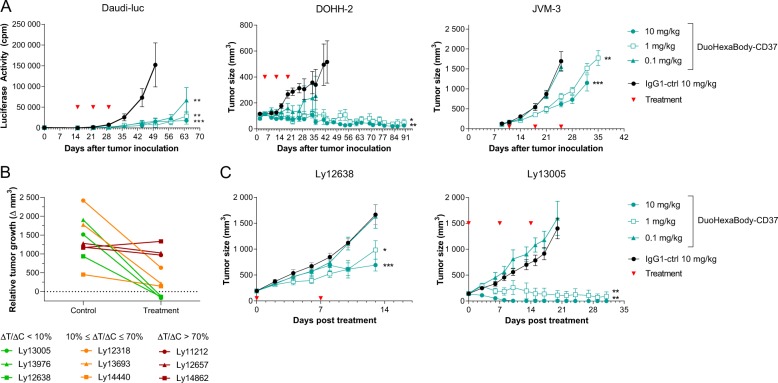
DuoHexaBody-CD37 shows anti-tumor activity in vivo in xenograft models. **a** The anti-tumor activity of DuoHexaBody-CD37 in vivo was evaluated in CDX models from Burkitt’s lymphoma (Daudi-luc), CLL (JVM-3) and B-cell lymphoma (DOHH-2) cell lines using SCID mice. IgG1-ctrl is a non-binding negative control antibody. Red arrows indicate antibody treatment (QWx3). Luciferase activity (mean ± standard error of the mean [SEM]) as a measure of tumor burden, or average tumor size (mean ± SEM) in mice treated with the indicated antibody dose is shown over time. **b** PDX screening in which tumor fragments from 9 NHL patients were subcutaneously implanted in SCID mice, using a one mouse per group design. Mice were dosed with either two weekly doses (QWx2) of 5 mg/kg DuoHexaBody-CD37 or PBS as a negative control. The relative tumor growth is presented as the difference in tumor volume between day of first treatment and day of analysis (7–25 days after initiation of treatment) in the DuoHexaBody-CD37-treated mouse (treatment, ΔT) and the control mouse (control, ΔC). The ratio between the relative tumor growth in the treatment and control mouse, specifically ΔT/ΔC, was used to categorize models as responders (ΔT/ΔC < 10%), intermediates (10% ≤ ΔT/ΔC ≤ 70%), or non-responders (ΔT/ΔC > 70%). **c** PDX dose response using two responding NHL models as described in **b** with indicated doses of DuoHexaBody-CD37 or IgG1-ctrl (*n* = 8 mice/group). Average tumor size is shown ±SEM. *P < 0.01, **P < 0.001, ***P < 0.0001.

The anti-tumor activity of DuoHexaBody-CD37 in vivo was evaluated in CDX models obtained by intravenous or subcutaneous injection of B-cell lymphoma-derived Daudi-Luc and DOHH-2 cells and CLL-derived JVM-3 cells, that express moderate to high levels of CD37 (Fig. 3e). First, we confirmed that DuoHexaBody-CD37 (which lacks cross-reactivity to murine CD37) has a normal clearance rate comparable to WT IgG1-ctrl in tumor-free mice, i.e. in the absence of target binding (data not shown). Next, SCID mice were injected with Daudi-luc, DOHH-2 or JVM-3 cells and treated with 0.1, 1, or 10 mg/kg DuoHexaBody-CD37 after tumors had established. Three weekly doses of DuoHexaBody-CD37 resulted in significantly reduced tumor growth in the Daudi-Luc model at all tested dose levels as compared to the IgG1-ctrl, and at 1 and 10 mg/kg in the JVM-3 and DOHH-2 models (Fig. 6a).

The anti-tumor activity of DuoHexaBody-CD37 was also assessed in PDX models, which offer more reliable results for clinical outcomes, because of their more conserved characteristics of the original tumor including heterogeneity, genetic and biological complexity and molecular diversity^37^. In a screening approach, we evaluated the anti-tumor efficacy induced by DuoHexaBody-CD37 using nine NHL PDX models in an experimental set up using a one mouse per group design. SCID mice were treated with two weekly doses of 5 mg/kg DuoHexaBody-CD37 or PBS after tumors had established, and the ratio between the relative tumor growth in the DuoHexaBody-CD37-treated mouse (ΔT) and the PBS control mouse (ΔC) was used to classify responders (ΔT/ΔC < 10%) versus non-responders (ΔT/ΔC > 70%) and intermediates (10% ≤ ΔT/ΔC ≤ 70%). Three models were classified as responders, three as intermediates and three as non-responders (Fig. 6b). From the three models that showed <10% relative tumor growth (i.e. tumor stasis or tumor regression), two models achieved complete tumor regression and did not grow out during the complete observation period (~60 days). Although CD37 mRNA expression was confirmed for all PDX models, variability between models was limited and could not be associated with response (data not shown). Follow-up cohort studies in two responding PDX models (Ly12638 and Ly13005) confirmed potent, dose-dependent anti-tumor activity of DuoHexaBody-CD37 at doses as low as 1 mg/kg (Fig. 6c). Collectively, these data indicate that DuoHexaBody-CD37 can mediate significant anti-tumor activity in both CDX and PDX in vivo models derived from different B-cell malignancy subtypes.

## Discussion

The treatment landscape for B-cell malignancies has rapidly evolved since initial approval of the CD20-targeting mAbs. With resistance to CD20-targeted therapies arising, a number of alternative B-cell surface antigens have been evaluated for targeted mAb therapy including CD19, CD22, CD30, CD79b, and CD37^38^. Similar to CD20, CD37 is highly expressed on tumor B cells across all major NHL and CLL subtypes. Although CD37 has long been recognized as a potential target for treatment of B-cell malignancies, it has recently received renewed interest with various agents currently in (pre)-clinical development. While multiple effector mechanisms have been reported for these agents, they are generally poor in inducing CDC^5–7^.

**Fig. 7:**
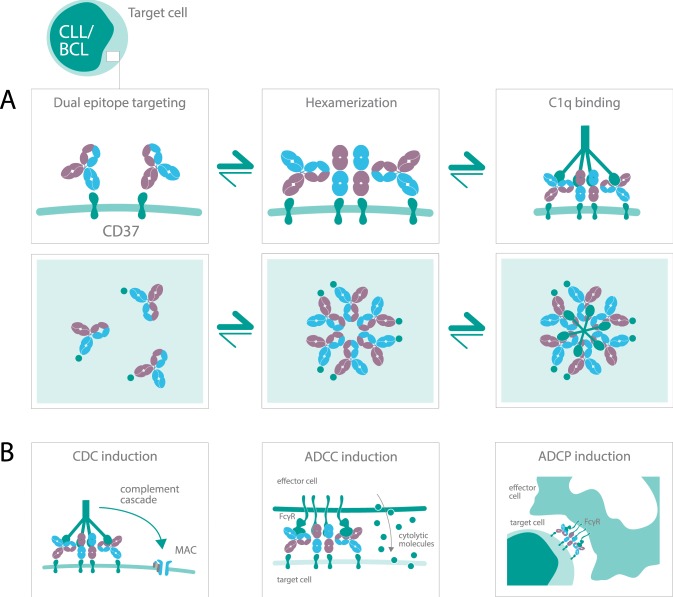
Mechanism of action of DuoHexaBody-CD37. **a** The biological process of DuoHexaBody-CD37 binding to CD37 receptors on the cell surface and the formation of hexameric antibody complexes, thereby providing an optimal docking site for C1q, the hexavalent first component of the complement system. Fc-Fc-mediated clustering of CD37 antibodies into hexameric complexes on the cell surface can be enhanced through the introduction of an E430G hexamerization-enhancing mutation and by dual epitope targeting. **b** DuoHexaBody-CD37 was shown to induce kill in malignant B cells by inducing highly potent CDC and through FcγR-mediated effector functions such as ADCC and ADCP.

Here, we introduce DuoHexaBody-CD37, a novel CD37-targeted agent generated using an innovative approach that combines DuoBody and HexaBody antibody platform technologies. DuoHexaBody-CD37 is a biparatopic bispecific IgG1 antibody with a hexamerization-enhancing mutation that induces strong anti-tumor activity in preclinical models in vitro and in vivo through potent CDC, ADCC and ADCP (Fig. 7). DuoHexaBody-CD37 exhibits highly potent CDC activity in vitro and ex vivo, which was superior to the parental WT or hexamerization-enhanced CD37 mAbs and to the combinations thereof. DuoHexaBody-CD37 not only outperformed all other CD37 antibody variants evaluated in ex vivo CDC assays using primary CLL patient samples, but also outperformed approved CD20 mAbs rituximab, ofatumumab and obinutuzumab. We demonstrated that the superior CDC efficacy of DuoHexaBody-CD37 is caused by enhanced antibody hexamerization upon target binding and by dual epitope targeting inherent to binding two non-overlapping epitopes on CD37. Locally increasing the density of Fc-domains through dual epitope targeting can potentiate CDC^36^ and may also favorably affect Fc-mediated antibody hexamerization, specifically in the context of the hexamerization-enhancing mutation. Consequently, the threshold for complement activation by DuoHexaBody-CD37 might be lower, which could result in enhanced anti-tumor efficacy in clinical settings. This could be of specific interest for patients who no longer respond to CD20 mAb treatment regimens, where antigen expression, cell surface antigen distribution or expression of complement inhibitors might be limiting factors^22^.

While DuoHexaBody-CD37 was shown to bind two non-overlapping epitopes on CD37, the fine mechanism of target binding, either bivalent or monovalent, and subsequent oligomerization into hexameric complexes remains to be elucidated. IgG oligomerization on target surfaces was recently reported to occur via Fc-Fc interaction-mediated recruitment of IgG molecules directly from solution, but also through lateral diffusion on the cell in case of preferentially monovalent binding IgG molecules^25^. CD37 is thought to be highly mobile due to its role in protein trafficking and organization in the plasma membrane and the formation of TEMs^39^. One could speculate that for DuoHexaBody-CD37 specifically, dual epitope targeting may optimize Fc-tail configuration in the process of IgG hexamerization, which may be augmented by lateral diffusion.

Besides inducing highly potent CDC, DuoHexaBody-CD37 was shown to efficiently engage with FcγRs in mediating ADCC and ADCP, indicating the mechanism of action of DuoHexaBody-CD37 is multifaceted. DuoHexaBody-CD37 compared favorably to rituximab in both FcγRIIIa and FcγRIIa crosslinking, in cell line models that showed comparable levels of CD20 and CD37 expression. Importantly, DuoHexaBody-CD37 efficiently depleted peripheral blood B cells, but not other leukocyte populations from healthy human whole blood. Furthermore, in xenograft models in vivo, DuoHexaBody-CD37 induced significant inhibition of tumor growth in a CLL CDX model, two NHL CDX models and six out of nine NHL PDX models. Notably, mice are not considered a suitable species to assess CDC-dependent tumor cell kill in vivo, suggesting FcγR-mediated effector functions may largely determine the observed anti-tumor activity^40^. Further studies are required to understand the contribution of individual effector mechanisms in vivo.

The current landscape of drug development in B-cell malignancies includes CD20-targeting antibodies such as ofatumumab and the glycoengineered obinutuzumab, and small molecule inhibitors targeting Bruton’s tyrosine kinase, Bcl-2 and PI3K-δ, such as ibrutinib, venetoclax, and idelalisib, respectively^3^. These growing numbers of therapeutic agents might also provide opportunities for combination therapy with CD37-targeting antibodies. Combinations of DuoHexaBody-CD37 with anti-CD20 antibodies could be of particular interest, as we have previously shown enhanced CDC in CLL and B-NHL primary patient cells with combinations of CD20 and CD37 mAbs containing hexamerization-enhancing mutations^33^. Furthermore, CD37 has been reported to contain ITIM-like and ITAM-like regulatory motifs that regulate pro-survival and pro-apoptotic signaling processes via the PI3K/AKT pathway^13^. We speculate that combinations of DuoHexaBody-CD37 with pro-apoptotic PI3K-δ-inhibitors such as idelalisib may work synergistically, however the role of DuoHexaBody-CD37 in apoptotic signaling via CD37 remains to be elucidated.

In summary, we present here a novel therapeutic antibody that, for the first time, combines the proprietary DuoBody and HexaBody antibody platforms to create a biparatopic CD37 IgG1 antibody with enhanced Fc-mediated hexamerization upon target binding. The potent anti-tumor activity exhibited by DuoHexaBody-CD37 in preclinical B-cell malignancy models highlights its therapeutic potential. Preparations to evaluate the clinical safety and preliminary efficacy of DuoHexaBody-CD37 in a first-in-human clinical trial are currently ongoing.

## Supplementary information

Supplementary Information
