# Supplementary material for: DuoHexaBody-CD37®, a novel biparatopic CD37 antibody with enhanced Fc-mediated hexamerization as a potential therapy for B-cell malignancies
Source: Blood Cancer J. 2020 Apr 28;10(3):30. doi: 10.1038/s41408-020-0292-7 (PMC7186228; doi:10.1038/s41408-020-0292-7)
Supplement: Supplementary file 1 — Supplementary Information [file 41408_2020_292_MOESM1_ESM.docx]

# Supplementary Information for

DuoHexaBody-CD37^®^, a novel biparatopic CD37 antibody with enhanced Fc-mediated hexamerization as a potential therapy for B-cell malignancies

Simone C. Oostindie^1,2^, Hilma J. van der Horst^3^, Laurens P. Kil^1^, Kristin Strumane^1^, Marije B. Overdijk^1^, Edward N. van den Brink^1^, Jeroen H.N. van den Brakel^1^, Hendrik J. Rademaker^1^, Berris van Kessel^1^, Juliette van den Noort^1^, Martine E. D. Chamuleau^3^, Tuna Mutis^3^, Margaret A. Lindorfer^4^, Ronald P. Taylor^4^, Janine Schuurman^1^, Paul W. H. I. Parren^1,2,5^, Frank J. Beurskens^1^, and Esther C. W. Breij^1†^

^1^Genmab, Utrecht, The Netherlands; ^2^Department of Immunohematology and Blood Transfusion, Leiden University Medical Center, Leiden, The Netherlands; ^3^Department of Hematology, Amsterdam University Medical Center, Amsterdam, The Netherlands; ^4^Department of Biochemistry and Molecular Genetics, University of Virginia School of Medicine, Charlottesville, Virginia, USA; ^5^Current affiliations: Department of Immunohematology and Blood Transfusion, Leiden University Medical Center, Leiden, The Netherlands and Lava Therapeutics, Utrecht, The Netherlands; ^†^Corresponding author

Correspondence to: [ebj@genmab.com](mailto:ebj@genmab.com)

**Supplementary Materials and Methods**

*Cells*

The (origin of) cell lines used in this study are summarized in Supplementary Table 1. All cell lines were routinely tested for mycoplasma contamination and generally aliquoted and banked to allow in vitro assays to be performed from frozen cells instead of continuously cultured systems to ensure authenticity of the cell lines. Commercially available purified primary chronic lymphocytic leukemia (CLL) cells from newly diagnosed patients were obtained from AllCells (Alameda, CA USA) and CLL peripheral blood mononuclear cells (PBMCs) were obtained from the Amsterdam University Medical Center (Amsterdam, The Netherlands) after written informed consent and stored using protocols approved by the Privacy Review Board of the Netherlands Cancer Registry in accordance with the declaration of Helsinki. PBMCs were isolated by density-gradient centrifugation (Ficoll-Paque PLUS, GE Healthcare, Chicago, IL, USA) from peripheral blood samples of lymphoma patients. Cells were used in experiments directly or stored in liquid nitrogen until further use.

*Antibodies*

Details on antibodies used to define cell subsets within flow cytometry-based CDC assays with primary CLL patient samples, ADCP assays and human whole blood assays are described in Supplementary Table 2-5.

*Alanine scanning*

A CD37 single residue alanine library (generated at GeneArt, Regensburg, Germany) was cloned into a pMAC expression vector and CD37 alanine mutants were expressed individually in FreeStyle HEK293F™ cells according to the manufacturer’s instructions (Thermo Fischer Scientific, Waltham, MA, USA). Antibody binding was determined as described in main text using 3 µg/ml AF488-conjugated monovalent binding variants (one irrelevant binding arm) of Hx-CD37-010 (bsIgG-CD37-010xctrl) and Hx-CD37-016 (bsIgG-ctrlxCD37-016) and expressed as the gMFI of the ungated cell population. The two non-cross-blocking test antibodies served as control antibodies for each other. To correct for expression differences between the different CD37 mutants, data were normalized against the gMFI of the control antibody, using the following equation (wherein ‘aa position’ refers to the particular position of the alanine substitution in the extracellular loop of the human CD37 mutant):

$${Normalized gMFI}_{aa position}={Log}_{10}\left( \frac{{gMFI}_{Test Ab}}{{gMFI}_{Control Ab}} \right)$$

To express loss or of antibody binding, a z-score (fold change in binding compared to binding of a control antibody) was determined according to the following calculation:

$$zscore \left( fold change \right)=\frac{{Normalized gMFI}_{aa position}- \mu}{\sigma}$$

Where μ and σ are the mean and SD of the normalized gMFI of all mutants. Z-scores < 0 are caused by loss of binding of bsIgG-ctrlxCD37-016 in comparison to bsIgG-CD37-010xctrl while z-scores > 0 are caused by loss of binding of bsIgG-CD37-010xctrl in comparison to bsIgG-ctrlxCD37-016. Amino acid residues where the z-score was higher than 1.5 (bsHx-CD37-010xctrl) or lower than –1.5 (bsHx-ctrlxCD37-016), indicated by the horizontal dotted lines, were considered as ‘loss of binding mutants’.

*ADCC assays*

Daudi target cells were labeled with 100 μCi ^51^Chromium (Amersham Biosciences, Uppsala, Sweden) and incubated with antibody concentration series and PBMCs from healthy human donors (isolated from buffy coats) as effector cells at a 100:1 effector to target ratio for four hours at 37°C. After incubation, the supernatant was transferred to Microscint-40 solution and released ^51^Chromium was counted in a scintillation counter (PerkinElmer, Waltham, MA, USA). Maximal and spontaneous lysis were determined using target cells incubated with 5% Triton X-100 or medium without effector cells, respectively. Specific lysis was calculated as (wherein cpm is counts per minute):

$$\% specific lysis=100* \frac{(cpm sample-cpm spontaneous lysis)}{(cpm maximal lysis-cpm spontaneous lysis)}$$

*Isolation of monocytes and culturing human monocyte-derived macrophages (h-MDM)*

PBMCs were isolated from a buffy coat from healthy donors (Sanquin) through centrifugation using LeucoSep^TM^-tubes (Greiner Bio-One, Alphen aan den Rijn, Netherlands) containing Lymphocyte Separation Medium (Corning). Human CD14^+^ monocytes were obtained from healthy donor PBMCs through positive isolation using CD14 MicroBeads (Miltenyi Biotec, Leiden, Netherlands) according to the manufacturer’s instructions. Monocytes were cultured in culture medium (CellGenix^®^ GMP DC serum-free medium with 50 ng/ml M-CSF) in Nunc™ dishes with UpCell™ surface (Thermo Fisher Scientific) at 37˚C/5%CO_2_ for 7-8 days to obtain human monocyte-derived macrophages (h-MDM). h-MDMs were characterized by flow cytometry for expression of myeloid- and macrophage-specific maturation markers (Supplementary Table 3).

*Animal husbandry*

In house animal experiments were performed in compliance with the Dutch animal protection law (WoD) translated from the directives (2010/63/EU) and, if applicable, the Code of Practice “animal experiments for cancer research” (Inspection V&W, Zutphen, The Netherlands, 1999) and were approved by the Ethical Committee of Utrecht. Daudi-luc and DOHH-2 studies were performed with female C.B-17/lcrHan^®^Hsd-*Prkdc*^scid^ mice, 7-8 weeks old, obtained from Envigo (Huntington, United Kingdom). Mice were housed in a barrier unit of the Central Laboratory Animal Research Facility (CLARF) of the Utrecht University (Netherlands) in sterile individually ventilated cages (IVC), 5 mice per cage, with sterile food and water provided ad libitum. Animals were housed and handled in accordance with good animal practice as defined by the Federation of European Laboratory Animal Science Associations (FELASA), in an Association for assessment and accreditation of laboratory animal care (AAALAC) and ISO 9001:2000 accredited animal facility (GDL, Utrecht, Netherlands).

JVM-3 studies were performed with CB17/ICR-*Prkdc*^SCID^/IcrlcoCrl mice (Vital River Laboratories, Beijing, China) at Crown Bioscience, China. All studies were conducted following an approved Institutional Animal Care and Use Committee (IACUC) protocol. All experimental data management and reporting procedures were in strict accordance with applicable Crown Bioscience, Inc. Guidelines and Standard Operating Procedures.

NHL patient-derived xenograft (PDX) studies (Supplementary Table 6) were performed with CB-17SCID mice (Janvier labs, Le Genest-Saint-Isle, France) at EPO Experimental Pharmacology & Oncology Berlin-Buch GmbH, Germany. All animal experiments were performed in accordance with the Guidelines for the Welfare and Use of Animals in Cancer Research and of the German Animal Protection Law, and approved by the local responsible authorities, following standard operating procedures for optimal performance.

*Cell line- and patient-derived xenograft studies*

Samples sizes of animal models were chosen based on the specific tumor growth properties (homogeneous vs heterogeneous tumor growth) and the expected efficacy of the drug. Furthermore, our experience has taught us that especially outliers will influence the statistical power. Therefore, 10-20% more mice are added to the study design in models where heterogeneous tumor growth is expected, such that animals with the highest and/or the lowest tumor volumes can be excluded from the study at the moment of randomization.

The Daudi-Luc cell line-derived xenograft (CDX) study was performed with 2.5x10^6^ cells inoculated intravenously (IV) into 7-8 weeks old CB-17 SCID mice. Based on bioluminescence imaging on day 0 (obtained within 2 hours post injection of Daudi-luc cells), the animals were randomized into groups of 9 animals each. Animals which showed no signal in the lungs at this time point and/or a clear signal at the injection site (tail vein) were excluded from randomization. At days 14, 21 and 28 after tumor cell inoculation, mice were treated with antibody by intraperitoneal (IP) injection. At weekly intervals, tumor growth was assessed using bioluminescence imaging on a Photon Imager (Biospace Lab, Nesles-la-Vallée, France). Before imaging, mice were anaesthetized via isoflurane and synthetic D-luciferin (BioThema, Handen, Sweden) was injected IP at a dose of 125 mg/kg; M3 Vision software (Biospace Lab, Nesles-la-Vallée, France) was used for image analysis. The DOHH-2 and JVM-2 CDX studies were performed with 1x10^6^ and 1x10^7^ cells inoculated subcutaneously (SC) into the flanks of 8-10 and 7-8 weeks old individual CB-17 SCID mice, respectively. Mice were randomized into groups (n=10) with equal tumor size distribution when tumors reached 100 and 200 mm^3^ respectively, and treated IP (DOHH-2) or IV (JVM-3) with 3 weekly doses (QWx3) of antibody. Tumor volume was measured at least twice per week using caliper measurements and calculated as 0.52 x (length) x (width)^2^. Animals were euthanized when tumors reached 1 500/2 000 mm^3^. During all CDX studies, heparinized blood samples were taken for determination of IgG levels in plasma. All CDX studies were performed using a blinded assessment of outcome measures.

NHL PDX models were performed with human tumor tissue implanted subcutaneously into the flanks of 6-7 weeks old CB17-SCID mice, and tumors were allowed to reach 100-250 mm^3^. Mice were randomized into groups (n=8) according to their tumor volume and treated IV with 2 weekly doses (QWx2) of antibody. Tumor volume was measured thrice weekly.

The PDX screening was performed as described above for regular PDX models, only using one mouse per group design. The relative tumor growth was defined as the ratio between the tumor growth in the DuoHexaBody-CD37-treated mouse (ΔT) and the control mouse (ΔC), specifically ΔT/ΔC. Tumor growth was calculated as the difference in tumor volume between day of first treatment and day of analysis, ideally between 7 and 25 days after initiation of treatment. Models in which the control mouse tumor volume did not increase 2-fold after start treatment were excluded from analysis. Models were categorized according to the following criteria: responders = ΔT/ΔC <10%; intermediates = 10% ≤ ΔT/ΔC ≤ 70%; non-responders = ΔT/ΔC > 70%. All PDX studies were performed using a blinded assessment of outcome measures.

**Supplementary Table 1. The (origin of) cell lines used in this study**

| Cell line | Origin | Company | Cat. No. |
| --- | --- | --- | --- |
| Daudi | Burkitt's lymphoma | ATCC | CCL-213 |
| DOHH-2 | Diffuse large B-cell lymphoma | DSMZ | ACC-47 |
| Jeko-1 | Mantle cell lymphoma | DSMZ | ACC-553 |
| JVM-2 | Mantle cell lymphoma | DSMZ | ACC-12 |
| JVM-13 | Mantle cell lymphoma | ATCC | CRL-3003 |
| OCI-Ly7 | Diffuse large B-cell lymphoma | DSMZ | ACC-688 |
| OCI-Ly19 | Diffuse large B-cell lymphoma | DSMZ | ACC-528 |
| Raji | Burkitt's lymphoma | ATCC | CCL-86 |
| Ramos | Burkitt’s lymphoma | ATCC | CRL-1596 |
| RC-K8 | Diffuse large B-cell lymphoma | DSMZ | ACC-561 |
| RI-1 | Diffuse large B-cell lymphoma | DSMZ | ACC 585 |
| SU-DHL-4 | Diffuse large B-cell lymphoma | ATCC | CRL-2957 |
| SU-DHL-8 | Diffuse large B-cell lymphoma | DSMZ | ACC-573 |
| U-2932 | Diffuse large B-cell lymphoma | DSMZ | ACC-633 |
| Wien-133 | Burkitt’s lymphoma | BioAnaLab, Oxford, UK |  |
| WIL2-S | EBV-positive B lymphoblastic cell line | ATCC | CRL-8885 |
| WSU-DLCL2 | Diffuse large B-cell lymphoma | DSMZ | ACC-575 |
| Z-138 | Mantle cell lymphoma | ATCC | CRL-3001 |

**Supplementary Table 2. Antibodies used for identification of cell subsets in primary patient sample CDC assays**

| **Target** | **Label** | **Target expression** | **Company** | **Cat. No.** |
| --- | --- | --- | --- | --- |
| CD45 | BV785 | Leukocytes | BioLegend | 304048 |
| CD45 | KO | Leukocytes | Beckman Coulter | B36294 |
| CD19 | PE | B cells | Beckman Coulter | A07769 |
| CD19 | PC7 | B cells | Beckman Coulter | IM3628 |
| CD3 |  | T cells | BD | 560365 |
| CD5 | APC | Expressed on most T cells and some B cell subsets, including some malignant B cells | BD | 345783 |
| CD5 | PE | See CD5-APC | DAKO | R084201 |

**Supplementary Table 3. Antibodies used for h-MDM characterization**

| Target | Label | Target expression | Company | Clone | Cat. No. |
| --- | --- | --- | --- | --- | --- |
| CD14 | PE-Cy7 | Maturation and lineage marker for monocytes/macrophages | BD Pharmingen | M5E2 | 557742 |
| CD11b | PE | General myeloid cell lineage and maturation marker | BD Pharmingen | ICRF44 | 555388 |
| CD64 | FITC | FcγRI (IgG1), expressed on mature antigen-presenting cells including macrophages | Biolegend | 10.1 | 305006 |
| CD80 | APC | B7-1, expressed on activated antigen-presenting cells, including macrophages | Miltenyi | 2D10 | 130-097-204 |
| CD163 | BV421 | Macrophage sub lineage/maturity marker | Biolegend | GHI/61 | 333612 |
| CD206 | BV711 | Mannose receptor, macrophage maturity/sub lineage marker | Biolegend | 15-2 | 321136 |
| FVS | eFluor660 | Staining of dead cells | BD Biosciences |  | 565694 |

**Supplementary Table 4. Antibodies used for identification of cell subsets in ADCP assays**

| Target | Label | Target expression | Company | Clone | Cat. No. |
| --- | --- | --- | --- | --- | --- |
| CD11b | PE | h-MDM | BD Pharmingen | ICRF44 | 555388 |
| CD19 | BV711 | Tumor B cells (Daudi) | Biolegend | SJ25C1 | 363026 |
| FVS | eFluor660 | Staining of dead cells | eBioscience |  | 65-0864-14 |

**Supplementary Table 5. Lineage-specific antibodies for identification of leukocyte subsets in human whole blood**

| Target | Label | Target expression | Company | Clone | Cat. No. |
| --- | --- | --- | --- | --- | --- |
| CD19 | BV711 | B cells | Biolegend | HIB19 | 302245 |
| CD3 | eFluor450 | T cells | e-Biosciences | OKT3 | 48-0037 |
| CD56 | PE-CF594 | NK cells | BD | NCAM16.2 | 564849 |
| CD16 | BV785 | Neutrophils | Biolegend | 3G8 | 302046 |
| CD66b | Pe-Cy7 | Granulocytes | Biolegend | G10F5 | 305115 |

**Supplementary Table 6. Overview of NHL PDX models used in this study**

| **Tumor model** | **Clinical type** | **Clinical status** | **Sample source** | **Cell-of-Origin (COO) classification** |
| --- | --- | --- | --- | --- |
| Ly11212 | Triple hit DLBCL | Relapsed | Peripheral blood | GCB* |
| Ly12318 | Double hit DLBCL | relapsed | Peripheral blood | ABC** |
| Ly12638 | LBCL | untreated | Solid biopsy | GCB |
| Ly12657 | pBCL | relapsed | Peripheral blood | intermediate/unclassified |
| Ly13005 | DLBCL | untreated | Solid biopsy | ABC |
| Ly13693 | DLBCL, EBV*** associated | untreated | Solid biopsy, retroperitoneal | GCB |
| Ly13976 | DLBCL | untreated | Solid biopsy, LN | non GCB |
| Ly14440 | DLBCL | relapsed | Solid biopsy, intraperitoneal | non GCB |
| Ly14862 | Double hit DLBCL | untreated | Solid biopsy | non GCB |

***** Germinal center B cell (GCB)

** Activated B-cell (ABC)

*** Epstein-Barr virus
